# The role of mitochondria in osteogenic, adipogenic and chondrogenic differentiation of mesenchymal stem cells

**DOI:** 10.1007/s13238-017-0385-7

**Published:** 2017-03-07

**Authors:** Qianqian Li, Zewen Gao, Ye Chen, Min-Xin Guan

**Affiliations:** 1 Division of Clinical Genetics and Genomics, The Children’s Hospital, Zhejiang University School of Medicine, Hangzhou, 310058 China; 2 Institute of Genetics, Zhejiang University and Department of Genetics, Zhejiang University School of Medicine, Hangzhou, 310058 China

**Keywords:** mesenchymal stem cells, mitochondria, differentiation

## Abstract

Mesenchymal stem cells (MSCs) are progenitors of connective tissues, which have emerged as important tools for tissue engineering due to their differentiation potential along various cell types. In recent years, accumulating evidence has suggested that the regulation of mitochondria dynamics and function is essential for successful differentiation of MSCs. In this paper, we review and provide an integrated view on the role of mitochondria in MSC differentiation. The mitochondria are maintained at a relatively low activity level in MSCs, and upon induction, mtDNA copy number, protein levels of respiratory enzymes, the oxygen consumption rate, mRNA levels of mitochondrial biogenesis-associated genes, and intracellular ATP content are increased. The regulated level of mitochondrial ROS is found not only to influence differentiation but also to contribute to the direction determination of differentiation. Understanding the roles of mitochondrial dynamics during MSC differentiation will facilitate the optimization of differentiation protocols by adjusting biochemical properties, such as energy production or the redox status of stem cells, and ultimately, benefit the development of new pharmacologic strategies in regenerative medicine.

## Introduction

Mesenchymal stem cells (MSCs) are multipotent cells originally found in the bone marrow that give rise to various specialized cells, including osteoblasts, adipocytes, and chondrocytes. MSC studies have progressed rapidly since the initial report of human MSCs isolation. These cells exhibit considerable promise for application in rebuilding damaged mesenchymal tissues (Chen et al., 2008b; Parekkadan and Milwid, 2010; Savkovic et al., 2014). So far, most MSC studies have focused on the activity of the nuclear genome; characteristics of mitochondrial activities have largely lagged behind. Mitochondria are essential organelles inside eukaryotic cells that are responsible for cellular energy production. Interestingly, several recent studies have shown variations in the abundances, morphology, and functions of mitochondria in different cell types that adapt to environmental and cellular cues (Collu-Marchese et al., 2015; Forni et al., 2016; Zhang et al., 2013). Here we summarize the current knowledge about the involvement of mitochondria in the stemness and differentiation progress of MSCs.

## Mitochondrial Properties are Modified During MSC Differentiation

Mitochondria are a distinguishing feature of eukaryotic cells, with an essential role in organismal longevity given their critical function in energy metabolism and cell death. In particular, the mitochondrion is one of the most sophisticated and dynamic responsive sensing systems in the cell. In addition, recent studies have proven that balanced mitochondrial dynamics and morphology are crucial to the maintenance of tissue homeostasis: mitochondrial dynamics regulates the fate of stem cells.

The significance of the perinuclear arrangement of mitochondria, which may be a characteristic feature of stem cells, is generally accepted. Taking MSCs as an example, the mitochondrial are mainly gathered around the nucleus in undifferentiated cells, whereas they are more uniformly distributed throughout the cytoplasm of differentiated cells (Hofmann et al., 2012; Quinn et al., 2013). In addition, the mitochondrial-to-cytoplasm area ratio increases during differentiation relative to that of undifferentiated cells (Forni et al., 2016; Lambertini et al., 2015). Researchers have confirmed that the area of individual mitochondria generally remains constant, which means that the increase in the mitochondrial-to-cytoplasm area ratio is not the result of mitochondrial swelling. Indeed, increased mitochondrial biogenesis is observed during both adipogenesis and osteogenesis, leading to an increased abundance of mitochondria in differentiated cells. It was shown that the level of the mitochondrial outer membrane protein TOM20 was greatly improved during adipogenic differentiation, and enhanced MitoTracker Green Staining further confirmed the increase in mitochondrial mass (Zhang et al., 2013). Correspondingly, mtDNA content was increased in differentiated cells (Wanet et al., 2014), and this increase was more striking in adipogenic cell differentiation (Forni et al., 2016). During osteogenesis, a dynamic change was revealed by qRT-PCR, with an initial decline upon induction, followed by a subsequent increase (Chen et al., 2008a). Furthermore, the morphology of mitochondria gradually becomes slender after the process of differentiation,and it has been shown that osteogenic differentiation of MSCs is accompanied by cristae development.

Studies have proven that the conditions of metabolic activity are different in MSCs from those of differentiated offspring cells: MSCs are more dependent on glycolysis, whereas differentiated cells depend more on oxidative metabolism (Hofmann et al., 2012; Hsu et al., 2016). Long-term cultured MSCs have been shown to exhibit a significantly lower proportion of cells with a perinuclear mitochondrial distribution, and these cells exhibit a higher ATP content and tendency to differentiate compared with cells from an earlier passage. Upon initiation of the differentiation process, mitochondria in MSCs are activated via a yet unknown mechanism and oxidative phosphorylation becomes the major source of ATP. This bioenergetic switch is especially important for osteogenic differentiation of MSCs as reported by Kowaltowski et al. (Tahara et al., 2009), and the ATP content of maturing cultures continuously increases along with nodule morphogenesis. Like osteogenic differentiation, oxygen consumption and the activities of respiratory enzyme complexes increase significantly during adipogenesis. In addition, Pietila et al. showed that red fluorescence of the probe JC-1 gradually became green along with the process of differentiation, indicating that mitochondrial membrane potential is reduced during the differentiation process (Pietila et al., 2012).

## The Tight Regulation of MSC Fate by ROS

Reactive oxygen species (ROS) are oxygen-derived small molecules, that react readily with various chemical structures including proteins, lipids, sugars, and nucleic acids. Mitochondria are the most important source of ROS within mammalian cells. For a long time, ROS have been considered to induce cellular dysfunction and organismal death via the destructive oxidation of intra-cellular components. In recent years, there has been an accumulating understanding of their role as signaling molecules (Atashi et al., 2015). Most research groups now believe that only unregulated levels of ROS are harmful, and a regulated basal level of ROS is necessary and advantageous for maintenance of cell functions, such as proliferation, differentiation, and survival (Sart et al., 2015; Wang et al., 2015). During the last decade, the impact of ROS on MSC differentiation has generated a great deal of interest due to its potential application in clinic.

Investigations have suggested the existence of a link between oxidative stress and impaired skeletal integrity. Increased intra-cellular levels of ROS were concluded to underline the observation that elder donor-derived MSCs exhibit a reduced potential for osteogenic differentiation (Tan et al., 2015), which is a pivotal pathogenetic mechanism of age-related bone and bone strength loss. Meanwhile, there is accumulating *in vitro* evidence suggesting that excess ROS impairs osteogenic differentiation. Studies have revealed that exogenous H_2_O_2_ reduced the activity of alkaline phosphatase, a marker of osteogenic differentiation, in culture (Lee et al., 2006; Tahara et al., 2009). Similarly, Chen et al. observed that the antioxidant enzymes SOD2 and catalase were significantly upregulated upon osteogenic differentiation, which led to a dramatic decrease in the intracellular ROS level (Chen et al., 2008a). In addition, by using cell viability assay, the researchers showed that differentiated cells were more resistant to exogenous ROS stress than undifferentiated cells. More recently, Kim et al. demonstrated that upregulation of ROS inhibits osteogenic differentiation of MSCs, in part through inhibition of the Hedgehog (Hh) signaling pathway, which is essential for bone development and maintenance (Kim et al., 2012).

Many research groups have focused on the osteogenic and adipogenic potential of aged MSCs. In general, MSCs from aged donors shift the balance in favor of adipocyte differentiation at the expense of osteoblast differentiation (Geissler et al., 2012). These results were consistent with the observations that ROS generation was increased during adipogenic differentiation. However, it is still controversial whether increased ROS is essential for adipocyte differentiation or it is a byproduct of the differentiation process. Yasunari et al. found that differentiation-inducing agents induced ROS generation in MSCs by an H_2_DCF assay, and the application of N-acetyl-L-cysteine (NAC) blocked adipogenic differentiation (Kanda et al., 2011). Tormos et al. revealed that ROS generated from mitochondrial complex III is required to initiate adipocyte differentiation by genetical manipulation of the complex (Tormos et al., 2011). In addition, these studies have shown that mitochondrial-targeted antioxidants could inhibit adipocyte differentiation, which was rescued by the addition of exogenous hydrogen peroxide. These results implied that increased ROS generation is not simply a consequence of adipocyte differentiation.

Although intensive research has been carried out on chondrogenic differentiation of MSCs, the role of ROS in chondrogenesis is less well characterized. Investigators have noticed that the ROS level was specifically high in developing chondrocytes of the embryonic limb, and chondrocyte maturation was accompanied by a progressive decrease in catalase activity in growing cartilage (Salas-Vidal et al., 1998; Schnabel et al., 2006). Regarding *in vitro* chondrogenic differentiation, ROS generation was increased during induction (Heywood and Lee, 2016; Jallali et al., 2007; Morita et al., 2007). Moreover, the differentiation markers of chondrocyte are upregulated by addition of H_2_O_2_, and chondrogenesis was inhibited by administration of antioxidant NAC, suggesting that ROS plays a critical role in chondrogenesis. Consistent with this, Kim et al. proved that ROS generated by Nox2 or Nox4 is essential for survival and differentiation in the early stage of chondrogenesis (Kim et al., 2010).

## Molecular Links of Mitochondrial Function and Dynamics with MSC Differentiation

**Figure 1:**
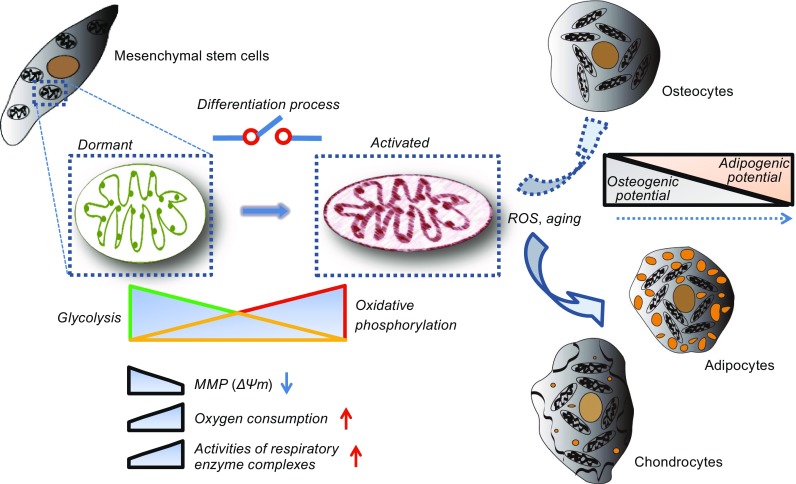
**The regulation of mitochondria dynamics and function is essential for successful differentiation of MSCs**. Mitochondria are mainly seen gathered around the nucleus in MSCs, whereas the mitochondria are more uniformly distributed in the cytoplasm of differentiated cells. Along with the process of differentiation, the morphology of mitochondria gradually becomes slender, and the number of mitochondria increases. The metabolic pattern has changed from glycolysis to oxidative phosphorylation; therefore, increased oxygen consumption and respiratory enzyme complex activation becomes logical. Notably, the membrane potential appears to be reduced in differentiated cells. The advancement of age and the high level of ROS can promote MSCs to adipocytes, whereas a low level of ROS can promote osteogenesis

There is a substantial body of evidence indicating that mitochondrial morphology and function are modulated during MSC differentiation. However, the molecular mechanisms linking mitochondrial dynamics with the regulation of differentiation are still poorly understood. It is believed that an orchestrated series of events are involved in MSC fate determination, for example, osteoblast differentiation requires the activation of many osteoblastgenic transcription factors (including Runx2, Osterix, and β-catenin) and inhibition of adipogenic transcription factors (PPARγ and CEBPα) (Chen et al., 2016). Growing evidence supports the bifunctional role of many transcription factors in the control of both nuclear and mitochondrial gene expression (Fig. 1).

The nuclear receptor peroxisome proliferator-activated receptor γ (PPARγ) is a crucial cellular and metabolic switch (Patel et al., 2014; Wan, 2010). Studies have shown that activation of PPARγ shifts the balance of MSCs fate by favoring adipocyte differentiation and inhibiting osteogenesis (Stechschulte et al., 2016; Wan, 2010). *In vitro* suppression of PPARγ enhanced osteogenesis and reduced adipogenesis. Consequently, PPARγ^+/−^ mice display a significantly higher bone mass due to increased bone generation (Akune et al., 2004). Huang et al. overexpressed PPARγ coactivator 1α (PGC-1α), which is the master regulator of mitochondrial biogenesis, in MSCs and observed a significant increase in genes related to mitochondrial functions and lipid metabolism (Huang et al., 2011). The over dose of PGC-1α prevented MSC differentiation into osteocytes upon induction; on the other hand, the adipogenic potential of the MSCs was increased. In addition, they found that PGC-1α knockdown inhibited adipocyte differentiation. Studies on PGC-1α have energized mitochondria research in stem cells biology, and have increased the chances that strategies to improve mitochondrial activity will be discovered.

Studies have demonstrated that mitochondria are maintained at a much lower activity state in MSCs compared with differentiated cells (Sanchez-Arago et al., 2013). Upon osteogenic induction, mitochondrial functions are upregulated to fulfill greater energy demand or facilitate other biochemical reactions that take place in cells. Wei et al. demonstrated that, upon osteogenic induction of MSCs, the levels of proteins involved in mitochondrial biogenesis are increased, including PGC-1α, mtTFA, DNA polymerase γ, enzymes of the TCA cycle, and protein subunits of respiratory enzymes (Hsu et al., 2016). Lambertini et al. found that nuclear factor of activated T cell complex 1 (NFATc1) is recruited to mtDNA and acts as a negative regulator of mtDNA transcription, which reveals the involvement of NFATc1 in the mineralization process (Lambertini et al., 2015). Sirtuins are protein deacetylases that are thought to play evolutionarily conserved roles in lifespan extension (Denu and Hematti, 2016). Recent studies have revealed that they also play roles in MSCs differentiation. SIRT1 is the most widely studied sirtuin, which deacetylates a number of substrates such as PGC-1α. It has been proven to be crucial for stem cell maintenance and differentiation (Min-Wen et al., 2016). The activation of SIRT1 impairs adipogenesis and enhances MSC osteogenesis. SIRT2 was found to regulate adipocyte differentiation through inhibition of PPARγ. Sirt3, Sirt5, and Sirt7 were also reported to be involved in mitochondrial biogenesis and the activation of mitochondrial function during adipogenic differentiation (Denu and Hematti, 2016). SIRT6 deficiency results in the impairment of differentiation potential of hMSCs into bone and cartilage (Pan et al., 2016).

Mitochondrial dynamics and their regulatory processes are supposed to be modulated during differentiation, leading to altered bioenergetic profiles. In the process of adipogenic differentiation and osteogenic differentiation, the content of citrate synthase increased obviously (Forni et al., 2016). Then, the expression of glycolytic enzymes and the yield of lactate were reduced during the osteogenic induction (Zhang et al., 2013). This suggests that the three carboxylic acid cycle process that occurs in the mitochondria is enhanced. Recently, Forni et al. showed that mitochondrial fusion proteins Mfn1 and Mfn2 were upregulated in the early stages of adipogenesis and osteogenesis, along with mitochondrial elongation (Forni et al., 2016). Regarding chondrogenesis, there was increased expression of fission proteins Drp1, Fis1, and Fis2. Knocking down of these genes resulted in a loss of differentiation ability. Moreover, enhanced mitophagy was observed during chondrogenesis. The authors claimed that the activation of mitophagy led to higher mitochondrial turnover during early chondrogenesis. Nuschke et al. found that accumulation of cleaved type II light chain 3 (LC3-II) protein, a marker for active autophagosomes, was correlated with osteogenic differentiation, indicating activation of autophagy upon stimulation of differentiation (Nuschke et al., 2014). Consistent with this conclusion, Song et al. recently reported that the adipogenic differentiation of MSCs was also promoted through the activation of autophagy (Song et al., 2015). Moreover, adipogenic differentiation could be blocked by the addition of autophagy inhibitors. Nonetheless, further studies are needed to better characterize the regulation of mitophagy during MSC differentiation.

## Hypoxia Influences MSC Differentiation

In the bone marrow, MSCs reside in a unique microenvironment (MSC niche). Recent advances have identified significant metabolic changes in the mitochondria that are regulated by environmental stimuli. Hypoxia, an important feature of MSC niche, has been proved to play an important role in maintaining stem cell fate, self-renewal, and multi-potency during the last decade. It was observed that there is an enhanced transcription and synthesis of glycolytic pathway enzymes and reduction of synthesis of proteins involved in mitochondrial catabolism in hypoxic cells. This holds true for MSCs as their proliferation, differentiation, and survival are affected by culture in low O_2_ tension. In mammalian cell, oxygen participates in reactions of aerobic energy synthesis as a substrate for cytochrome oxidase, the terminal enzyme of mitochondrial respiratory chain. In hypoxia, decreased mitochondrial size and reduced mitochondrial mean velocity have been observed (Varela-Rey et al., 2009). Mitochondrial morphology is associated with HIF-1α stabilization. Moreover, mitochondria act as O_2_ sensors, and contribute to the cell redox potential, ion homeostasis, and energy production. Specifically, hypoxia could reduce mitochondrial fusion by impairing mitochondrial membrane potential, which in turn could induce supercomplexes disassembly, increasing ROS production.

Evaluation of adipocyte lineage-specific transcripts and osteocyte lineage-specific transcripts showed that the expression of ALPL in MSCs in severe hypoxia is higher than in normoxia, indicating MSCs in hypoxia are more prone to osteogenic differentiation than in normoxia (Ejtehadifar et al., 2015). The expression of osteogenesis-related genes, such as alkaline phosphatase, Type I collagen, and osteocalcin was significantly increased under hypoxia (Boyette et al., 2014). Hypoxia suppressed adipogenesis and associated HIF1α and PPARG gene expression in hMSCs and enhanced osteogenesis and associated HIF1A and RUNX2 gene expression (Wagegg et al., 2012; Zhang et al., 2013). Moreover, shRNA-mediated knockdown of HIF-1α suppressed hypoxia-induced osteogenesis (Wagegg et al., 2012). The mechanism was demonstrated that HIF-1α can suppress oxidative metabolism through inhibition of pyruvate dehydrogenase (PDH) by PDH kinase (PDK) and activate the expression of glycolytic enzymes (Papandreou et al., 2006). On the other hand, the mitochondrial electron transport chain deficiency made the expression of HIF-1α severely reduced, and HIF-1 DNA binding was diminished, which suggested that electron transport chain activity is required for activation of HIF-1 (Agani et al., 2000).

So far, most hypoxia studies showed that long term culture of MSCs in hypoxia promotes a genetic program maintaining their undifferentiated and multipotent status. However, the degree and duration of hypoxia described in the literatures vary greatly and may result in very different effects on the proliferation and differentiation capacities of MSC. Future work will continue to explore hypoxia-induced effects and help to position mitochondrial function, dynamics, and signaling within MSC differentiation. And, more remarkable, we cannot draw conclusions that *in vitro* culture in hypoxia mimics the niche until we understand the *in vivo* signature of MSCs.

## Perspectives

Throughout the last decade, rapid progress has been made in the area of stem cells and metabolism. Various studies suggest that mitochondrial activity or dormancy plays a role in maintaining the undifferentiated state of MSCs, whereas proper activation is essential for successful differentiation. In addition, mitochondrial ROS are reported to not only regulate the differentiation process but also contribute to the direction determination of differentiation. However, the understanding of the role played by mitochondria during stem cell differentiation is still in its early stages, and a deeper knowledge of the interaction between mitochondria dynamics and MSC differentiation is needed. Indeed, a series of studies are working on these important open questions: What are the mechanisms restricting mitochondrial biogenesis and favoring a glycolytic metabolism in MSCs? How can activated mitochondrial biogenesis favor various differentiation processes? What are the molecular players linking mitochondrial dynamics and the differentiation processes? Do noncoding RNAs decode the interplay between mitochondria and cell differentiation? Taken together, the data we have summarized here clearly indicate a critical role for mitochondria dynamics during MSCs differentiation. We believe this rapidly moving field promise many more to come, and progress in this research will facilitate the optimization of *in vitro* differentiation protocols, which can finally benefit the better design of cell engineering using MSCs.
